# Supplementary material for: Psycho-social problems among older people residing in community of Chitwan, Nepal—A cross-sectional study
Source: PLoS One. 2024 Sep 19;19(9):e0310849. doi: 10.1371/journal.pone.0310849 (PMC11412642; doi:10.1371/journal.pone.0310849)
Supplement: Attachment — Submitted filename: Response to Reviewers.docx [file pone.0310849.s001.docx]

18^th^ August, 2024

Kamlesh Kumar Sahu
Academic Editor
PLOS ONE

PONE-D-24-12655 “Psycho-Social Problems among Older People Residing in Community of Chitwan, Nepal - A Cross-sectional Study”

Dear Editor

Thank you very much for your suggestions and giving me an opportunity to correct article. We also thank the reviewers for their substantial time, efforts and generous comments on our manuscript. We have carefully revised the manuscript in response to the extensive, valuable, and insightful comments we received. We tried to address all in point-to-point response.

In the revised manuscript we corrected grammatical errors, and incorporated all the suggestions. Please find the list of suggestions along with our responses to each point in the appended below.

We hope that this revised manuscript will be suitable for publication in Plos one.

Yours Sincerely,

Sunita Poudyal

**Subject: Responses to Reviewers**

| **SN** | **Questions** | **Responses** |
| --- | --- | --- |
|  | **Reviewer #1:** |  |
| (1) | Avoid the term "elderly" as well as being ageist, it is an under-inclusive term and therefore uses the term older people/persons. | It has been addressed in corrected version.  The term elderly is replaced with use of term older people/persons/ senior citizen in the manuscript. |
| (2) | The introduction is also loaded with an ageist and negative view of old age, as a process of decline, multi morbidity, etc. | Ageist is replaced with the older people in the introduction and written as:  The number of older people is increasing dramatically around the world, including in Nepal. |
| (3) | The methodology should consider whether the scales have been previously validated in older people in Nepal.  The proposed descriptive analysis can be included in a first phase and then a mutivariate analysis should be carried out. | The scales used in this study have been previously validated in older people in Nepal.  A mutivariate analysis (Regression) wasn’t done as it is a cross sectional study. If it is needed, we will do it. |
|  | **Reviewer #2:** |  |
| (1) | References need through check as per the journal style there few mistakes. | References and citations were checked thoroughly and managed according to journal style. |
| (2) | Conclusion need to be in separate heading | Conclusion was kept in a separate heading. |
| (3) | In the objective 'This study aimed to find out the psycho-social problems among older people residing in Bharatpur, Chitwan.' in place of Bharatpur, Chitwan you may write community | In the objective 'This study aimed to find out the psycho-social problems among older people residing in community. |
|  | **Reviewer #3:** |  |
| (1) | Community will be better than writing a city name in objective  References need to recheck and correct as per the journal style. | The city name was replaced by word community.  References rechecked and corrected as per the journal style. |
| (2) | Conclusion needs to be in a separate heading | Conclusion was kept in a separate heading. |
| (3) | Term "elderly" as well as being ageist terms are not inclusive terms therefore use the term older people/persons. | The term elderly was replaced with use of term older people/persons/ senior citizen. |
|  | **Overall from Academic Editor** |  |
|  | We note that the grant information you provided in the ‘Funding Information’ and ‘Financial Disclosure’ sections do not match. When you resubmit, please ensure that you provide the correct grant numbers for the awards you received for your study in the ‘Funding Information’ section. | It is revised in the funding information and financial disclosure section as  “This study is funded by Nepal Health Research Council as Provincial Health Research Grant FY 2079/080. Grant receiver: Ms Sunita Poudyal. The funder had no role in study design, data collection and analysis, decision to publish and preparation of manuscript” |
|  | Please provide a complete Data Availability Statement in the submission form, ensuring you include all necessary access information or a reason for why you are unable to make your data freely accessible. | Data availability statement  All data are fully available without restriction in Open Science Framework  **Registration DOI**  <https://doi.org/10.17605/OSF.IO/HTGYD> |
|  | PLOS requires an ORCID iD for the corresponding author in Editorial Manager. | Sunita poudyal: ORCID iD- 0000-0001-9062-9651  Kalpana Sharma: 0000-0001-8761-8505  Hem Kumari Subba: 0000-0003-2692-3607 |
|  | Please review your reference list to ensure that it is complete and correct. If you have cited papers that have been retracted, | References and citations are complete and correct.  We have made some changes:   - References with more than six authors, listed the first six author names, followed by “et al.” - Added PMID and PMC number in references. |
|  | Please amend the title either on the online submission form or manuscript | According to suggestion, it is corrected in the online submission also |
|  |  |  |

Once again thank you very much for your time and suggestion on the manuscript.
